# Ipsilateral hypoperfusion caused by intracerebral steal phenomenon after carotid artery stenting: a case report

**DOI:** 10.1186/s12883-021-02208-6

**Published:** 2021-05-08

**Authors:** Zhizhong Yan, Zhonghua Shi, Yuhai Wang, Chunlei Zhang, Huize Liu, Jin Cai, Xin Zhang

**Affiliations:** 1 Department of Neurosurgery, The 904 th Hospital of the Joint Logistics Support force of Chinese People’s Liberation Army, No. 101 North Xinyuan Road, Jiangsu Province 214000 Wuxi, People’s Republic of China; 2 Department of Neurosurgery, Jinling Hospital, The First School of Clinical Medicine, Southern Medical University, No. 305 Zhongshan East Road, Jiangsu Province 210002 Nanjing, People’s Republic of China

**Keywords:** Intracerebral steal phenomenon, Stenting, Endovascular treatment, Carotid artery

## Abstract

**Background:**

Abnormal hypoperfusion on the surgical side after carotid artery stenting is rare. Neurological deterioration caused by it is deceptive, which can easily lead to misdiagnosis. The mechanism of hypoperfusion has rarely been demonstrated. We present here a fully studied case with a high probability of intracerebral steal phenomenon.

**Case presentation:**

A 68-year-old male with severe right internal carotid artery stenosis and left internal carotid artery occlusion underwent right stenosis stent implantation. Restlessness and left limb hemiplegia occurred within 24 h after the procedure, which was similar to hyperperfusion syndrome. However, postoperative computerized tomography perfusion (CTP) revealed abnormal hypoperfusion in the right hemisphere. Transcranial Doppler (TCD) also showed decreased flow velocity in the right middle cerebral artery, and increased flow velocity in the right anterior cerebral artery. We considered that intracerebral steal phenomenon might be the cause, then hypervolemic therapy was accepted and the symptoms completely resolved after 3 days.

**Conclusions:**

Ipsilateral hypoperfusion is rarely seen after carotid artery stenting. Intracerebral steal phenomenon may be the underlying mechanism. CTP or TCD is helpful for the early detection of this adverse event.

## Background

Internal carotid artery (ICA) stenosis is one of the main factors for stroke, and patients with contralateral internal carotid artery occlusion are associated with a higher rate of stroke [1]. The treatment of these patients is challenging, and carotid artery stenting (CAS) is currently recommended for these patients with a greater surgical risk [2]. Perioperative complications such as infarction, hyperperfusion, and cerebral hemorrhage are relatively common [3]. Here, we report a rare postoperative complication in this kind of patient. Hypoperfusion appeared on the surgical side after CAS, which resulted in neurological deterioration similar to hyperperfusion syndrome. We suppose that intracerebral steal phenomenon may be a strong candidate for its underlying mechanism.

## Case presentation

**Fig. 1:**
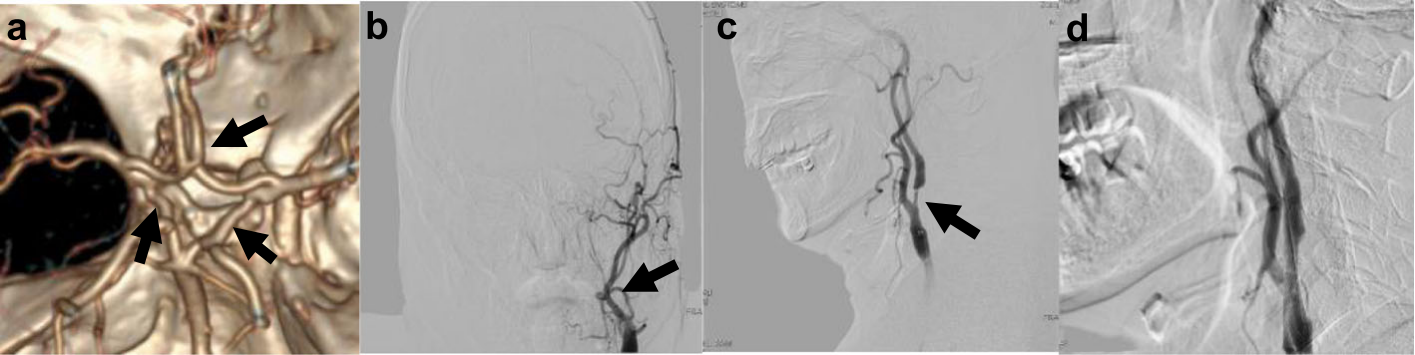
**a** Anterior communicating artery and bilateral posterior communicating artery are well developed (arrow).** b** DSA shows left ICA occlusion (arrow). **c **DSA shows right ICA severe stenosis (arrow). **d **DSA shows right ICA following carotid artery stenting

**Fig. 2:**
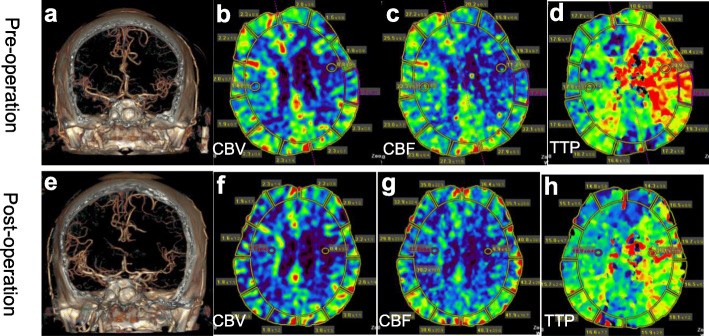
**a** and **e** CTA before and 24 hours after stenting show the intracranial vessels are similar. **b**, **c**and **d **CTP before operation.** f**, **g** and **h** CTP 24 hours after operation

A 68-year-old male presented to the outpatient clinic complaining of drowsiness and fatigue for 2 weeks. He had a history of left-sided cerebral infarction 5 months ago and no hypertension, diabetes, coronary artery disease or atrial fibrillation. Since then, dual antiplatelet and statin therapy was taken and there was no transient ischemic attack. Neurological examination showed right limb dyskinesia along with aphasia. The admission low-density lipoprotein cholesterol was 1.64 mmol/L, preoperative coagulation was normal with prothrombin time (PT) of 11.9 s, activated partial thromboplastin time (APTT) of 27.3 s, international normalized ratio (INR) of 1.04, thromboelastography arachidonic acid (AA) inhibition rate of 96 % and adenosine diphosphate (ADP) inhibition rate of 90 %. The electrocardiogram showed sinus tachycardia. Because of claustrophobia, only computed tomography (CT) images could be provided. After admission, one-stop computed tomography angiography (CTA)/computed tomography perfusion (CTP) was performed. CTA showed that the Willis circle was well developed (Fig. 1a). CTP showed that cerebral blood volume (CBV) and cerebral blood flow (CBF) in the left hemisphere were lower than those in the right hemisphere, while time to peak (TTP) was significantly higher (Fig. 2b, c and d).

Digital subtraction angiography (DSA) revealed occlusion at the beginning of the left ICA and 75 % stenosis in the right ICA according to the North American Symptomatic Carotid Endarterectomy Trial criteria (Fig. 1b and c). The right ICA stent (XACT 6–8*40 mm, Abbott) implantation was performed after DSA (Fig. 1d). During the procedure, there was no significant decrease in blood pressure or heart rate, nor any neurological deterioration. Unfortunately, 8 h after CAS, the patient developed restlessness, accompanied by a marked increase in blood pressure (170/100 mmHg). After the exclusion of intracranial hemorrhage by CT, sedation and antihypertensive treatment were used, and the blood pressure was controlled below 120/80 mmHg. However, the patient developed left limb hemiplegia 24 h after the procedure. Then one-stop CTA/CTP was performed again. Intracranial vessels were comparable to that before CAS (Fig. 2a and e). CTP showed CBV and CBF in the left hemisphere were improved and TTP was reduced significantly. But CBV and CBF in the right hemisphere were not only lower than pre-operation, but also lower than that in the left hemisphere (Fig. 2f, g and h). Dynamic Transcranial Doppler (TCD) was also performed. Flow velocity of the right middle cerebral artery (MCA) decreased 24 h after CAS, while that of the right anterior cerebral artery (ACA) increased significantly (Table 1). We supposed that hypoperfusion in the right hemisphere might be caused by left-to-right blood theft. Then hypervolemic treatment (intravenous infusion of saline 3000 ml per day) was accepted and the blood pressure was maintained above 140/90 mmHg. After 3 days, the patient’s restlessness and left limb hemiplegia were completely relieved. No recurrence of cerebral ischemia occurred during 3 months of telephone follow-up.

**Table 1:** TCD results before, 24 h and 3 days after CAS

Vm (cm/s)	Pre-operation	24 h after CAS	3 days after CAS
R-ICA	164.4	39.5	28.7
L-ICA	--	--	--
R-MCA	50.7	46.5	72.5
L-MCA	17.8	24.6	23.2
R-ACA	30.2	97.8	68.4
L-ACA	27.5	38.5	37.3

*Abbreviations: Vm *mean blood flow velocity

## Discussion and conclusion

Carotid artery occlusion occurs in 5–15 % of patients with carotid artery stenosis [4]. In recent years, CAS has been increasingly used as an alternative for patients at high risk of carotid endarterectomy, including those with carotid artery occlusion [5]. Hyperperfusion, cerebral hemorrhage and stroke are common perioperative complications, however, postoperative hypoperfusion on the CAS side is rarely reported.

There was no neurological deterioration during and after procedure, so infarction caused by intraoperative emboli could be excluded. In patients with carotid artery occlusion, the flow velocity of ipsilateral and contralateral MCA increased significantly in the early stage after CAS [6]. And Sadato et al. also found that CBF in both hemispheres increased after unilateral CAS [7]. However, in this case, the flow velocity of MCA decreased, and hypoperfusion appeared on the treated side after CAS. We consider that the occurrence of hypoperfusion is probably caused by left-to-right intracerebral steal phenomenon.

Intracerebral steal phenomenon is a pathological process in which a large amount of blood flows in reverse through a vascular bed with lower resistance, resulting in reduced blood supply of other adjacent arteries and corresponding clinical symptoms [8]. This phenomenon has been mainly reported in subclavian artery occlusion or arteriovenous malformations [9]. In this case, preoperative ischemia in the left hemisphere was more severe than that in the right side according to CTP, so the capillary in the left hemisphere may be extremely dilated. When the right ICA stenosis was relieved, more blood flow may pass through the anterior communicating artery into the left hemisphere with lower resistance, resulting in hypoperfusion on the right side.

Although there are few reports about intracerebral steal phenomenon, it may not be uncommon, mainly because its symptoms are similar to other common complications, which can easily lead to misdiagnosis. If intracerebral steal phenomenon can’t be detected in time, it may lead to ischemia aggravation, or even infarction. Dynamic TCD monitoring in the early stage after CAS may be helpful. If the MCA flow velocity on the treated side decreases abnormally after CAS, the existence of intracerebral steal phenomenon should be highly suspected.

In conclusion, hypoperfusion caused by intracerebral steal phenomenon is a rare complication after CAS. It can cause neurological deterioration, but symptoms are deceptive. Postoperative dynamic TCD monitoring and perfusion CT are helpful for early detection to prevent this harmful side effect.

## Data Availability

The data used and analyzed during the present study are available from the corresponding author on reasonable request.

## Abbreviations

CT: Computed tomography
CTP: Computed tomography perfusion
CTA: Computed tomography angiography
CBV: Cerebral blood volume
CBF: Cerebral blood flow
TTP: Time to peak
TCD: Transcranial doppler
DSA: Digital subtraction angiography
ICA: Internal carotid artery
MCA: Middle cerebral artery
ACA: Anterior cerebral artery
CAS: Carotid artery stenting

## References

[1] AbuRahma AF, Metz MJ, Robinson PA (2003). Natural history of > or = 60 % asymptomatic carotid stenosis in patients with contralateral carotid occlusion. Ann Surg.

[2] Kokkinidis DG, Chaitidis N, Giannopoulos S, Texakalidis P, Haider MN, Aronow HD, Giri JS, Armstrong EJ (2020). Presence of contralateral carotid occlusion is associated with increased periprocedural stroke risk following CEA but not CAS: a meta-analysis and meta-regression analysis of 43 studies and 96,658 patients. J Endovasc Ther.

[3] Ederle J, Dobson J, Featherstone RL, Bonati LH, van der Worp HB, de Borst GJ, Lo TH, Gaines P, Dorman PJ, Macdonald S (2010). Carotid artery stenting compared with endarterectomy in patients with symptomatic carotid stenosis (International Carotid Stenting Study): an interim analysis of a randomised controlled trial. Lancet.

[4] Faggioli G, Pini R, Mauro R, Freyrie A, Gargiulo M, Stella A (2013). Contralateral carotid occlusion in endovascular and surgical carotid revascularization: a single centre experience with literature review and meta-analysis. Eur J Vasc Endovasc Surg.

[5] Ricotta JJ 2nd, Upchurch GR Jr, Landis GS, Kenwood CT, Siami FS, Tsilimparis N, Ricotta JJ, White RA. The influence of contralateral occlusion on results of carotid interventions from the Society for Vascular Surgery Vascular Registry. J Vasc Surg. 2014;60(4):958–64. discussion 964 – 955. 10.1016/j.jvs.2014.04.036 25260471

[6] Yan Z, Yang M, Niu G, Zhang B, Tong X, Guo H, Zou Y (2019). Hemodynamic surveillance of unilateral carotid artery stenting in patients with or without contralateral carotid occlusion by TCD/TCCD in the early stage following procedure. Front Neurol.

[7] Sadato A, Maeda S, Hayakawa M, Adachi K, Toyama H, Nakahara I, Hirose Y (2018). Carotid stenting for unilateral stenosis can increase contralateral hemispheric cerebral blood flow. J Neurointerv Surg.

[8] Wang D, Li Z, Zheng X, Cong H, Zhang T, Wang Z, Wang Y, He J (2020). Head and neck CT angiography to assess the internal carotid artery stealing pathway. BMC Neurol.

[9] Randhawa N, Squires JP, Heran MKS, Mann SK (2017). Where did the clot go? An unusual complication of mechanical thrombectomy caused by malignancy related subclavian steal phenomenon in a patient with acute basilar artery occlusion. J Neurointerv Surg.

